# Acellular pertussis vaccines effectiveness over time: A systematic review, meta-analysis and modeling study

**DOI:** 10.1371/journal.pone.0197970

**Published:** 2018-06-18

**Authors:** Ayman Chit, Hossein Zivaripiran, Thomas Shin, Jason K. H. Lee, Antigona Tomovici, Denis Macina, David R. Johnson, Michael D. Decker, Jianhong Wu

**Affiliations:** 1 Leslie Dan Faculty of Pharmacy, University of Toronto, Toronto, ON, Canada; 2 Sanofi Pasteur, Swiftwater, PA, United States of America; 3 Department of Mathematics and Statistics, York University, Toronto, ON, Canada; 4 Dalla Lana School of Public Health, University of Toronto, Toronto, ON, Canada; 5 Sanofi Pasteur, Toronto, ON, Canada; 6 Sanofi Pasteur, Lyon, France; 7 Department of Health Policy, Vanderbilt University, Nashville, TN, United States of America; Public Health England, UNITED KINGDOM

## Abstract

**Background:**

Acellular pertussis vaccine studies postulate that waning protection, particularly after the adolescent booster, is a major contributor to the increasing US pertussis incidence. However, these studies reported relative (ie, vs a population given prior doses of pertussis vaccine), not absolute (ie, vs a pertussis vaccine naïve population) efficacy following the adolescent booster. We aim to estimate the absolute protection offered by acellular pertussis vaccines.

**Methods:**

We conducted a systematic review of acellular pertussis vaccine effectiveness (VE) publications. Studies had to comply with the US schedule, evaluate clinical outcomes, and report VE over discrete time points. VE after the 5-dose childhood series and after the adolescent sixth-dose booster were extracted separately and pooled. All relative VE estimates were transformed to absolute estimates. VE waning was estimated using meta-regression modeling.

**Findings:**

Three studies reported VE after the childhood series and four after the adolescent booster. All booster studies reported relative VE (vs acellular pertussis vaccine-primed population). We estimate initial childhood series absolute VE is 91% (95% CI: 87% to 95%) and declines at 9.6% annually. Initial relative VE after adolescent boosting is 70% (95% CI: 54% to 86%) and declines at 45.3% annually. Initial absolute VE after adolescent boosting is 85% (95% CI: 84% to 86%) and declines at 11.7% (95% CI: 11.1% to 12.3%) annually.

**Interpretation:**

Acellular pertussis vaccine efficacy is initially high and wanes over time. Observational VE studies of boosting failed to recognize that they were measuring relative, not absolute, VE and the absolute VE in the boosted population is better than appreciated.

## Introduction

The highly contagious pathogen *Bordetella pertussis* is estimated to cause 16 million cases of whooping cough (pertussis) and result in 195,000 pediatric deaths around the world every year [1]. Vaccination is the most effective measure to reduce the burden of disease. In the US, vaccine programs introduced in the 1940s were associated with a drop in the rate of disease from 150/100,000 to 1/100,000 by the 1970s [2]. The rate of reported pertussis, however, recently reached national levels of 9/100,000 in 2010 and then 15/100,000 in 2012, eliciting concern from public health experts on the effectiveness of the current pertussis control program [2]. In 2012, Washington State, among others in the US, experienced an epidemic of pertussis, with county-level incidence soaring as high as 415/100,000, and unexpectedly high incidence in 13 and 14 year-old adolescents [3]. Similarly, the outbreak reported in California in 2014 saw county-level incidence rates as high as 142/100,000, with particular peaks in numbers of cases among children 7–10 years of age, and among adolescents 13–17 years of age. Of particular concern was the fact that a high proportion of the reported cases were in children and adolescents who were up to date on their vaccination series.

These epidemics raised public health concerns and prompted investigations into the vaccine effectiveness (VE) of the childhood acellular pertussis series (5-dose DTaP) and the full acellular pertussis series (5-dose DTaP + 1-dose Tdap) in states experiencing outbreaks. Notably, Misegades et al investigated the VE of a 5-dose series of DTaP in a cohort of children living in California in 2010. They reported an initially high VE of 98.1% (95% CI: 96.1 to 99.1) in the first 12 months that gradually declined to 71.2% (45.8 to 84.8) 60 months or longer after the fifth DTaP dose [4]. In a 2012 study, Acosta et al. examined VE after a sixth dose of Tdap administered at 11 years of age to adolescents living in Washington State. VE was reported as 73.1% (60.3% to 81.8%) for the first 12 months, and 34.2% (-0.03% to 58.0%) 24–47 months after receipt of Tdap [5]. Acosta et al concluded that Tdap provided only moderate protection in the first year after vaccination, and that effectiveness waned rapidly thereafter by approximately 35% annually. The authors also postulated that the waning protection likely was a major contributor to the increasing pertussis incidence among adolescents. Similar concerns about Tdap VE were raised by other researchers investigating outbreaks in Wisconsin and California [6,7].

While these studies clearly indicated that the efficacy of acellular pertussis vaccination declined over time, the concern that VE after boosting with Tdap is moderate and wanes at an alarming rate may be overstated. To appreciate the absolute VE against pertussis after the sixth dose of the acellular pertussis series (Tdap), one must first consider immunization history with DTaP. We know from Misegades et al. that children with five doses of DTaP still have 71% absolute protection against pertussis by the time they turn 10 years of age. In the Acosta et al study, all participating children (including reference group participants) had received five doses of DTaP and would have had residual protection against pertussis when they became eligible for Tdap on their 11^th^ birthday. As such, the Acosta study actually measured protection offered by a Tdap booster relative to a reference group that had an estimated 71% protection against pertussis from their DTaP series, even if they did not receive the 6^th^-dose Tdap booster. A Tdap booster administered to these children therefore provides an additional 73.1% worth of protection, rather than merely 73.1% worth of absolute protection.

In this study we aim to integrate empirical evidence in the published literature to estimate the absolute VE of acellular pertussis vaccines. To do so, we conduct a systematic review of acellular pertussis VE studies. We then use modeling techniques to derive the absolute protection offered by acellular pertussis vaccines over time.

## Methods

### Systematic review

Using the PRISMA frame work, we conducted a systematic review of the literature to identify vaccine effectiveness or efficacy studies (both abbreviated as VE) of acellular pertussis vaccines [8]. In our review we applied a number of inclusion and exclusion criteria; first, we included studies of US licensed acellular pertussis vaccines used as per the US immunization schedule. Second, the study had to examine a 5-dose DTaP childhood series administered by 5 years of age (referred to herein as the primary acellular pertussis series), or a primary acellular pertussis series followed by an adolescent Tdap booster administered between 11–18 years (referred to herein as the full acellular pertussis series). We excluded studies focusing on adult vaccination such as maternal immunization and cocooning. Third, only studies reporting VE over discrete time periods after a vaccine series were included. Consequently, we excluded studies that only reported VE over a defined risk period for individuals that received their immunization at different times prior to the risk period. Finally, only studies reporting VE against disease were included. Studies of immune responses were therefore excluded.

These criteria were applied to first screen titles and abstracts, studies not excluded at this stage went on to full text review where the same exclusion criteria were assessed again. Two independent reviewers (TS, JL) completed initial screening based on titles and abstracts, and then full text review of articles that passed screening. A working group (AC, HZ, AT, DM, JW) was consulted to arbitrate any issues regarding study eligibility.

We searched the academic literature, up to August 31st, 2017, using Embase, Medline, Scopus, and Web of Science. Our search algorithm consisted of combinations of keywords, MeSH terms, and topic headings germane to *“pertussis*,*” “effectiveness*,*” “comparative effectiveness*,*”* and *“efficacy”* (S1 Fig). Several iterations of the search were conducted from November 2015 to January of 2016 for sensitivity optimization. Foreign articles were translated using Google translate.

We assessed publication bias by using funnel plots with p-values from Egger’s test [9]. To assess quality we used a modified Blacks and Down checklist for randomized and non-randomized studies [10].

### Meta-analysis and modeling

Our objective was to estimate absolute VE (i.e compared to vaccine naïve children) against pertussis over time offered by: 1) the primary acellular pertussis series (5-dose DTaP), and 2) the full pediatric acellular pertussis series (5-dose DTaP + 1-dose Tdap booster).

We employed mathematical transformations in instances where the empirical literature did not present data we could directly pool to meet our objectives. All transformations preserved the underlying variance of the original distributions. The published studies reported measures of association as Odds Ratios (OR), Relative Risk (RR) or Hazard Ratios (HR). Given that pertussis is a rare event (probability <10%), when applicable, we assumed that all these measures of association were equivalent [11]. Heterogeneity among effect measures was assessed by years since vaccination utilizing Higgin’s I^2^. When pooling measures of association by strata (i.e. years since vaccination), we utilized a random-effects model with DerSimonian-Laird estimators [12].

#### Primary acellular pertussis series

Only one study by Misegades et al. estimated the absolute protection offered by the primary acellular pertussis series, over a number of years [4]. Based on this study, we defined the absolute reduction in the risk of pertussis at a given age *n*, as *RR*_*PS*,*n*_ where:

$${RR}_{PS,n}=\frac{\mathrm{Risk}\;\mathrm{of}\;\mathrm{pertussis}\;\mathrm{at}\;n\;\mathrm{years}\;\mathrm{of}\;\mathrm{age}\;\mathrm{after}\;\mathrm{primary}\;\mathrm{acellular}\;\mathrm{pertussis}\;\mathrm{series}}{\mathrm{Risk}\;\mathrm{of}\;\mathrm{pertussis}\;\mathrm{at}\;n\;\mathrm{years}\;\mathrm{of}\;\mathrm{age}\;\mathrm{in}\;\mathrm{an}\;\mathrm{unvaccinated}\;\mathrm{group}}$$

It follows that absolute vaccine protection one year after completion of the primary acellular pertussis series (at six years of age) is defined as *RR*_*PS*,6_.

All other studies of the primary acellular pertussis series estimated waning in vaccine protection using the first year after series completion as a reference. We defined the results of these studies as ${\hat{RR}}_{PS,n}$, where:

$${\hat{RR}}_{PS,n}=\frac{\mathrm{Risk}\;\mathrm{of}\;\mathrm{pertussis}\;\mathrm{at}\;n\;\mathrm{years}\;\mathrm{of}\;\mathrm{age}\;\mathrm{after}\;\mathrm{primary}\;\mathrm{acellular}\;\mathrm{pertussis}\;\mathrm{series}}{\mathrm{Risk}\;\mathrm{of}\;\mathrm{pertussis}\;\mathrm{at}\;6\;\mathrm{years}\;\mathrm{of}\;\mathrm{age}\;\mathrm{after}\;\mathrm{primary}\;\mathrm{acellular}\;\mathrm{pertussis}\;\mathrm{series}}$$

We transformed ${\hat{RR}}_{PS,n}$ data into a time series of absolute reductions in risk (*RR*_*PS*,*n*_) using the following equation:

$${RR}_{PS,n}={\hat{RR}}_{PS,n}\times \;{RR}_{PS,6}$$

The transformation using the scalar *RR*_*PS*,6_ was applied to the entire distribution of ${\hat{RR}}_{PS,n}$ thus preserving the variance.

We then evaluated the presence of study heterogeneity (e.g. Higgin’s I^2^) and pooled the *RR*_*PS*,*n*_ data from the various studies by year since series completion.

Pooled *RR*_*PS*,*n*_ values were converted to *VE*_*PS*,*n*_ estimates using the following equation:

$${VE}_{PS,n}=({1-RR}_{PS,n})\times 100\%$$

Next, the *VE*_*PS*,*n*_ data was fit to an exponential decay model in order to estimate the average waning rate (i.e. λ) across time calculated using the following equation:

$${VE}_{expected}={VE}_{baseline}e^{-\lambda (time)}$$

This was used to forecast protection in additional years beyond the empirical data and to estimate the rate of VE waning over time.

#### Full acellular pertussis series

All the studies of the full pediatric acellular pertussis series relied on a reference group of individuals with a primary acellular pertussis series, and as such, they reported relative measures of vaccine protection. We defined the results of these studies as *RR*′_FS,n_ where:

$${RR\prime }_{\mathrm{FS,n}}=\frac{\mathrm{Risk}\;\mathrm{of}\;\mathrm{pertussis}\;at\;n\;years\;of\;age\;\mathrm{after}\;\mathrm{full}\;\mathrm{pediatric}\;\mathrm{acellular}\;\mathrm{pertussis}\;\mathrm{series}\;}{\mathrm{Risk}\;\mathrm{of}\;\mathrm{pertussis}\;at\;n\;\mathrm{years}\;\mathrm{of}\;\mathrm{age}\;\mathrm{after}\;\mathrm{primary}\;\mathrm{acellular}\;\mathrm{pertussis}\;\mathrm{series}}$$

Similar to the primary acellular pertussis series, we evaluated the presence of study heterogeneity (e.g. Higgin’s I^2^) and pooled the appropriate *RR*′_FS,n_ data from the various studies by year since vaccination. All *RR*′_FS,n_ values were converted to measures of relative VE (*rVE*_FS,n_) defined as:

$${rVE}_{\mathrm{FS},n}=(1-\;{RR\prime }_{\mathrm{FS},n})\times 100\%$$

We then converted *rVE*_FS,n_ to absolute VE (vs. a vaccine naïve population) using the following equation:

$${VE}_{\mathrm{FS},n}=1-(1-r{VE}_{\mathrm{FS},n}\;)(1-{VE}_{\mathrm{PS},n})$$

All *VE*_FS,n_ data was fit to an exponential decay curve in order to estimate the average waning rate (i.e. λ) across time.

The uncertainty surrounding the conversion of ${\hat{RR}}_{PS,n}$ into an absolute risk reduction (i.e. *RR*_*PS*,*n*_) was examined through probabilistic sensitivity analyses (PSA). Given the nature of vaccine effectiveness and relative risk (i.e. 0%≤VE≤100%), we utilized a beta distribution with shape parameters α and β to generate a posterior distribution [i.e. *p*(*π*|*y*,*α*,*β*)]. This was achieved using the method of moments approach and the delta method (to calculate the standard error (SE) for *RR*_*PS*,6_). The following formulas describe how we derived of the shape parameters.

$$\alpha ={RR}_{PS,6}\left(\frac{{RR}_{PS,6}(1-{RR}_{PS,6})}{\left({SE}^{2}\right)}-1\right)$$

$$\beta ={(1-RR}_{PS,6})\left(\frac{{RR}_{PS,6}(1-{RR}_{PS,6})}{\left({SE}^{2}\right)}-1\right)$$

Utilizing the shape parameters, we generated the posterior distribution over a 1000 iterations. This provided a range of values describing the variability of *RR*_*PS*,6_. Specifically, the first and third quartiles (i.e. q1, q3) were identified as values characterizing the 25^th^ and 75^th^ percentile of the distribution. These values (i.e. q1, q3) were applied as conversion factors to illustrate the variability associated with *RR*_*PS*,6_.

## Results

Our systematic review yielded a total of seven publications, which are summarized in Table 1 [4,5,6,7,13,14,15]. Three of these studies described the VE of the primary acellular pertussis series, and four the VE of the full acellular pertussis series (S2 Fig). We scored the studies between Good and Fair on the Downs and Black critical appraisal tool, and we did not identify any bias through the Egger’s test (S3 Fig).

**Table 1:** Summary of publications included in model.

Author [ref]	Vaccine	Study Location	Sample Size	Period of Investigation	Study Design	Clinical Case Definition	Statistical Model	Quality Score	Quality Rating
Misegades et al [4]	DTaP primary series only	California, USA	682 cases 2,016 controls	2010	Case-control study with vaccine naïve controls estimating absolute VE after DTaP primary series over time	Definition of the CSTE*	Logistic regression	17	Fair
Klein et al [13]	DTaP primary series only	California, USA	277 cases 3,318 controls	2006–2011	Case-control study. All subjects received DTaP primary series. Study estimates waning of VE over time using year 1 after DTaP as a reference	Patients testing PCR positive for pertussis	Logistic regression	21	Good
Tartof et al [14]	DTaP primary series only	Minnesota & Oregon, USA	Cohorts: 224,378 (MN) 179,011 (OR) Cases: 458 (MN); 89 (OR)	1998–2003	Cohort study. All subjects received DTaP primary series. Study estimates waning of VE over time using year 1 after DTaP as a reference	Definition of the CSTE	Log binomial model	16	Fair
Liko et al [15]	DTaP primary series followed by Tdap booster	Oregon, USA	Cohort: 958,330 Cases: 709	2012	Cohort study. All subjects including reference group received DTaP primary series. Study estimates incremental VE over time from Tdap booster	Definition of the CSTE	Not disclosed	17	Fair
Acosta et al [5]	DTaP primary series followed by Tdap booster	Washington, USA	450 cases 1,246 controls	2012	Case-control study. All subjects including reference group received DTaP primary series. Study estimates incremental VE over time from a Tdap booster	Definition of the CSTE	Conditional logistic regression	23	Good
Koepke et al [6]	DTaP primary series followed by Tdap booster	Wisconsin, USA	Cohorts: 225,130 (Full) 225,130 (Full) Cases: 940	2012	Cohort study. All subjects including reference group received DTaP primary series. Study estimates incremental VE over time from Tdap booster	Definition of the CSTE	Poisson regression	19	Good
Klein et al [7]	DTaP primary series followed by Tdap booster	California, USA	340 cases 3,841 controls	2006–2015	Cohort study. All subjects including reference group received DTaP primary series. Study estimates incremental VE over time from Tdap booster	Patients testing PCR positive for pertussis	Cox regression	19	Good

*CSTE: Council of State and Territorial Epidemiologists

Two of the primary acellular pertussis series studies examined VE in California and focused on the 2010 outbreak, while the third examined VE in Minnesota and Oregon over the period 1998 to 2003 [4,13,14]. Misegades et al. was the only study to estimate VE in comparison to a vaccine naïve population [4]. The other two studies did not enroll any vaccine naïve children. Instead, they assessed the decline in VE over time by using the first year after the primary acellular pertussis series as a reference [13,14].

The VE of the full acellular pertussis series was studied in the states of Oregon, Washington, Wisconsin and California. All studies examined VE during the 2012 outbreak, with the California study also considering the 2014 outbreak [15,5,7,6]. None of the studies included a group of vaccine naïve children. Thus, they estimated the additional protection offered by completing the full pediatric acellular pertussis series above residual primary acellular pertussis series protection [5,6,7].

**Fig 1:**
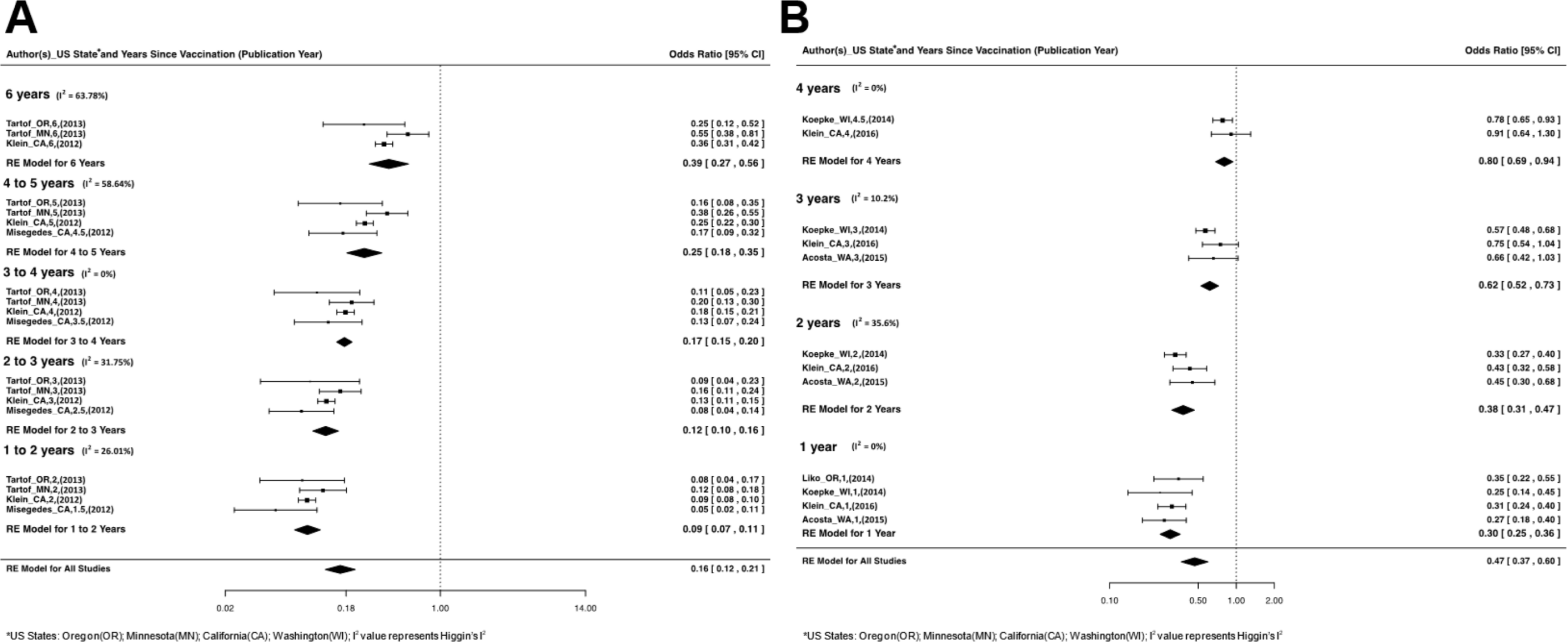
Meta-analysis of acellular pertussis vaccines. This figure represents the relative risk (OR) of pertussis infection occurring at different time points after vaccination. The I2 values (i.e. Higgin’s I2) quantifies the proportion of heterogeneity and dispersion in the meta-analytic model. Panel A: Represents a Meta-analysis of the OR of pertussis infection when comparing children immunized with the primary acellular pertussis series (5-dose DTaP) compared to vaccine naïve children. These OR values in Panel A were the product of a mathematical transformation of relative (5-dose DTaP) OR values. Panel B: Represents a meta-analysis of the OR of pertussis infection when comparing children immunized with the full acellular pertussis series (5-dose DTaP and 1-dose Tdap) compared to children immunized with only the primary acellular pertussis series.

We found that the primary acellular pertussis series substantially reduces the absolute risk of pertussis compared to no vaccine, with a relative risk (RR) of 0.09 (95% CI: 0.07 to 0.11) in the first 2 years after series completion. Protection declines over time reaching a RR of 0.39 (95% CI: 0.27 to 0.56) after 6 years of series completion. Children, who completed their full series by 11 years of age, received considerable added protection against pertussis from their Tdap booster. Compared to children that only received the primary acellular pertussis series, the RR of pertussis was 0.30 (95%CI: 0.25 to 0.36) 1 year after the Tdap booster. The added benefit of the booster was retained four years out with a RR of 0.8 (95%CI: 0.69 to 0.94). Fig 1 provides more detail on the meta-analysis by year since vaccination.

**Fig 2:**
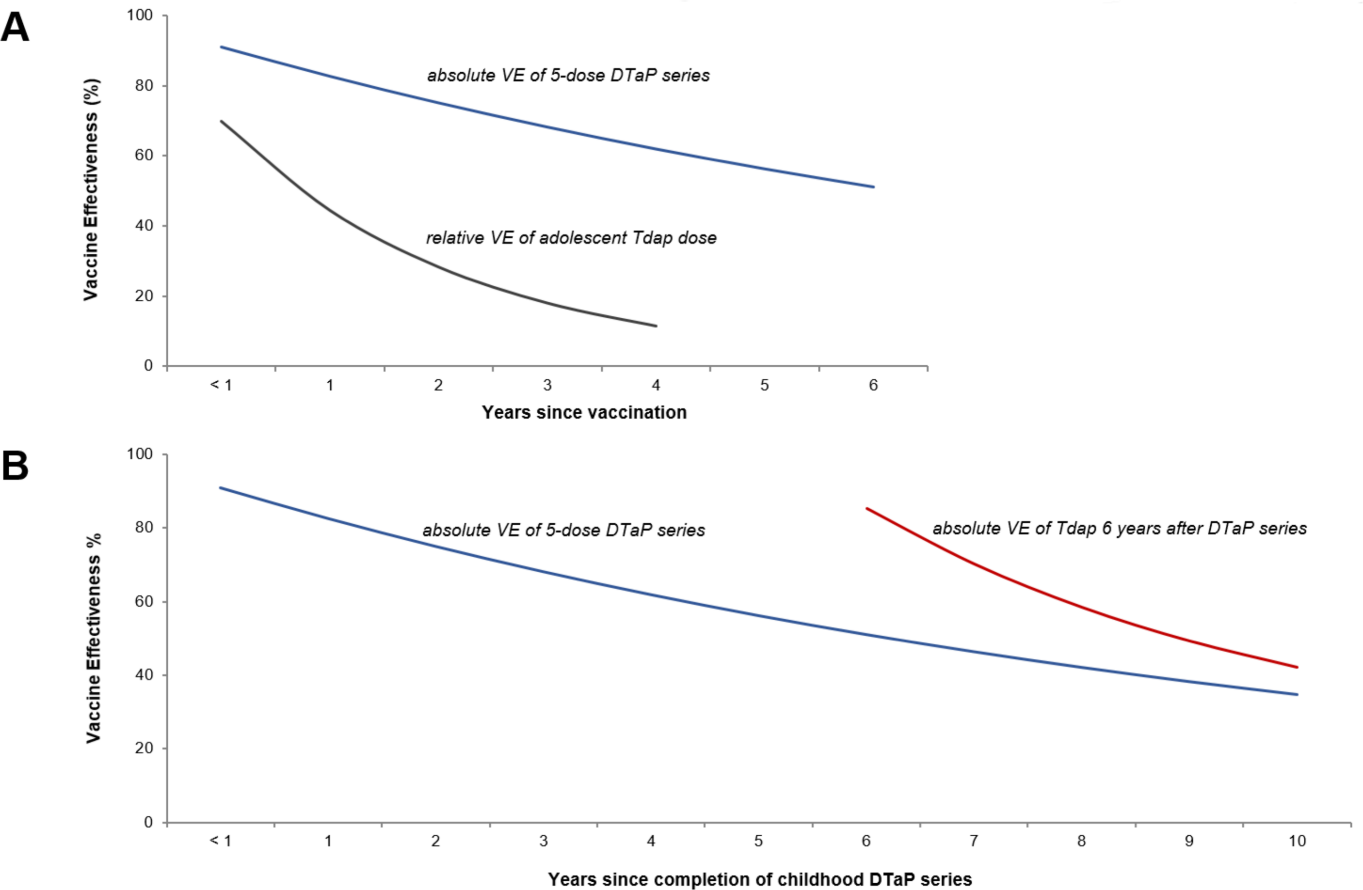
Acellular pertussis vaccine effectiveness (VE) over time. Panel A: The blue line represents the absolute VE (ie, vs a pertussis vaccine naïve population) of the 5-dose DTaP childhood series. The grey line represents the relative VE (ie, vs DTaP vaccine-primed population) of 1-dose Tdap vaccine given in early adolescence, as reported by previous researchers. Panel B: The blue line again represents the absolute VE of the primary acellular pertussis series. The red line represents the modeled absolute VE of the full acellular pertussis series (ie, 1 dose of Tdap vaccine given in early adolescence 6 years after completion of the DTaP childhood series).

Next we translated these data into VE estimates against pertussis. The primary acellular pertussis series absolute VE was estimated at 91% (95% CI: 87% to 95%) and declined by 9.6% per year. The relative VE of boosting with Tdap compared to only receiving the primary acellular pertussis series was 70% (95% CI: 54% to 86%) and declined by 45.3% per year. Based on these data, we estimated the absolute VE of the full acellular pertussis series to be 85% (95%CI: 84% to 86%) and to decline by 11.7% (95% CI: 11.1% to 12.3%) per year. As such, by 3 years, 5 years, and 7 years post the full acellular pertussis series, the absolute protection against pertussis is expected to be 49% (95%CI: 48% to 50%), 37% (95%CI: 35% to 37%), and 28% (95%CI: 27% to 29%). Fig 2 provides a graphical representation of these data.

In the sensitivity analysis *RR*_*PS*,*n*_ ranged between 0.21 (VE = 79%) and 0.10 (VE = 90%). Meta-analysis plots are presented in online supplement 4 (S4 Fig). Furthermore, the downstream effect of conducting this sensitivity analysis showed waning rates ranging between *λ*_*VE*,90%_ = 4% and *λ*_*VE*,79%_ = 17%.

PRISMA checklist attached as supplement (S1 Checklist)

## Discussion

The age-specific distribution of cases in recent outbreaks in the US has led to investigations into the VE of the adolescent 6^th^-dose acellular pertussis vaccine booster. Three US VE studies that ensued raised concerns about initial VE after this booster, and about the decline of VE over time [5,6,7]. Acosta et al. reported an initial VE of 73% that declined to 34% over the subsequent two to four years [5]. Similarly, Koepke et al. reported an initial VE of 75% that declined to 12% in the subsequent four years [7]. Finally, Klein et al. reported a VE of 69% and 9%, after one, and four years respectively from receiving the series [6].

In this study we show that the absolute VE of the full 6-dose acellular pertussis series is better than appreciated and can be estimated at 85% (95%CI: 84% to 86%) in the first year after series completion and declines by 11.7% per year. By 18 years of age the full pediatric acellular vaccine series is expected to offer an absolute protection against pertussis of 28.2% (95%CI: 27% to 29%).

In contrast to other recent reviews of pertussis vaccine effectiveness, our review is the first to tackle duration of protection over time from both the primary series and the booster dose. Fulton et al [16] conducted the most recently published review of pertussis vaccine effectiveness. The researchers focused on vaccine effectiveness with in a three year period after completion of the primary series and did not provide estimates of the degree of waning in protection over time. McGirr et al authored the only other review that examined waning of vaccine protection over time after the primary series; however, they did not examine waning after the booster dose [17]. Further, both Fulton and McGirr, in their meta-analysis, did not strictly examine the current US schedule which is most relevant for clinical decision making in the US today.

In our literature search we identified only one study by Haller et al. examining absolute VE offered by a full pediatric acellular pertussis vaccine series [18]. However, this publication did not qualify for our meta-analysis and modeling study as it was conducted in Germany and did not follow the US immunization schedule. Regardless, Haller et al.’s work provides an important point of comparison. The researchers examined the VE of a 4-dose DTaP completed by 14 months of age followed by a Tdap booster between 9 and 17 years of age. As a unique feature not present in any of the US studies, the researchers were able to estimate absolute VE directly as they were able to identify a reference group of vaccine naïve adolescents. They found that the absolute VE of the full acellular pertussis series was 96.5% (95% CI: 88.3% to 98.7%). This result supports our finding that initial protection after the full acellular pertussis series is higher than generally appreciated in recent studies conducted in the US.

The findings of our study should not take away from the broader concerns regarding the recent epidemiology of pertussis. This epidemiology is complex and influenced by a large set of factors that are difficult to measure and relate to one another [19]. As such, substantial research is still needed to improve our understanding and to inform public health responses. Chief amongst these is further development of robust mathematical disease models. To date several modeling studies have been published that illustrate the complex system behavior that once taken together can provide a more nuanced view of the recent resurgence of pertussis [20,21,22,23,24,25]. However, further modeling and empirical research is required to understand the changing pertussis epidemiology and we hope that our analysis provides some additional clarity on the role that acellular pertussis vaccines play in stemming the rise of pertussis disease in this complex system.

Our analysis has limitations that should be noted. First, none of the studies included in our study were randomized in nature. Non-randomized studies have inherent limitations that make them susceptible to biases and residual confounding. Second, we assumed that protection waned exponentially over time. While this assumption is in line with biological theory [26], further vaccine efficacy estimates would be required to confirm the robustness of this assumption. Third, our analysis focused on the outcome of pertussis disease. As such we could not directly infer to what degree the acellular vaccines provide protection against transmission. Fourth, we only considered studies in the US because of the considerable difference in vaccination schedules around the world. Finally, the Downs and Black methodology was used to assess quality of reporting, internal validity (bias and confounding), power, and external validity of this study. The published studies scored either “Fair” or “Good”, and the highest score was 23 out a possible 30 points. As such this points to the need for further higher quality studies in this field.

In conclusion, acellular pertussis VE is initially high and wanes over time. A combination of further empirical work and mathematical modeling research are needed to improve our understanding of the drivers behind recent pertussis outbreaks in the US.

## Supporting information

S1 Checklist PRISMA checklist. (DOC)

S1 Fig Summary of systematic review search strategy. (TIFF)

S2 Fig Results of systematic review. (TIFF)

S3 Fig Funnel plots. (TIFF)

S4 Fig Sensitivity analysis methods and results. (TIFF)
