# Supplementary figures and images for: Acellular pertussis vaccines effectiveness over time: A systematic review, meta-analysis and modeling study
Source: PLoS One. 2018 Jun 18;13(6):e0197970. doi: 10.1371/journal.pone.0197970 (PMC6005504; doi:10.1371/journal.pone.0197970)

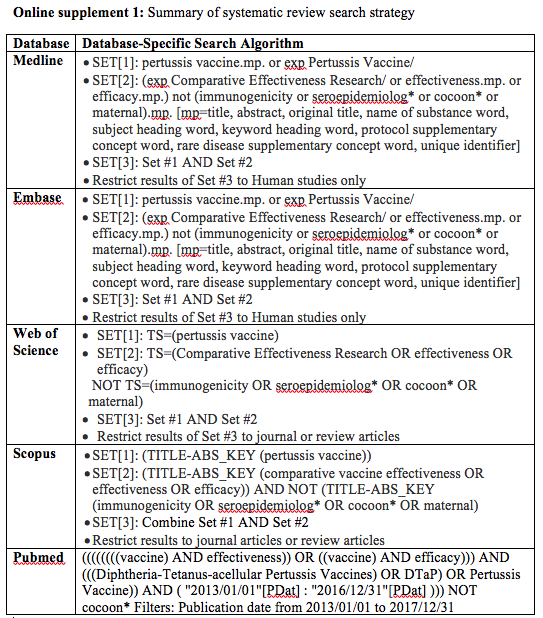

Supplement: S1 Fig — (TIFF) [file pone.0197970.s002.tiff]

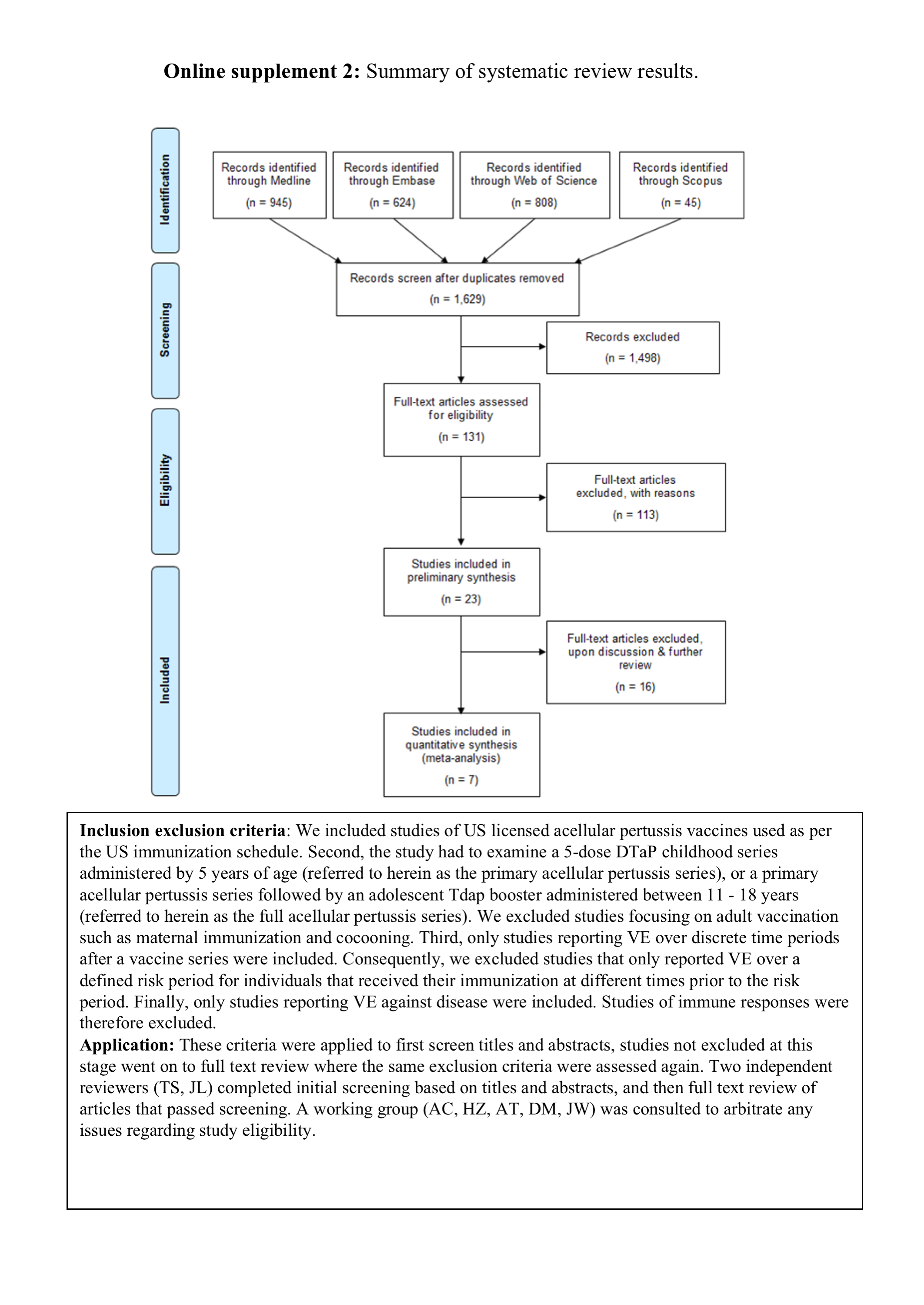

Supplement: S2 Fig — (TIFF) [file pone.0197970.s003.tiff]

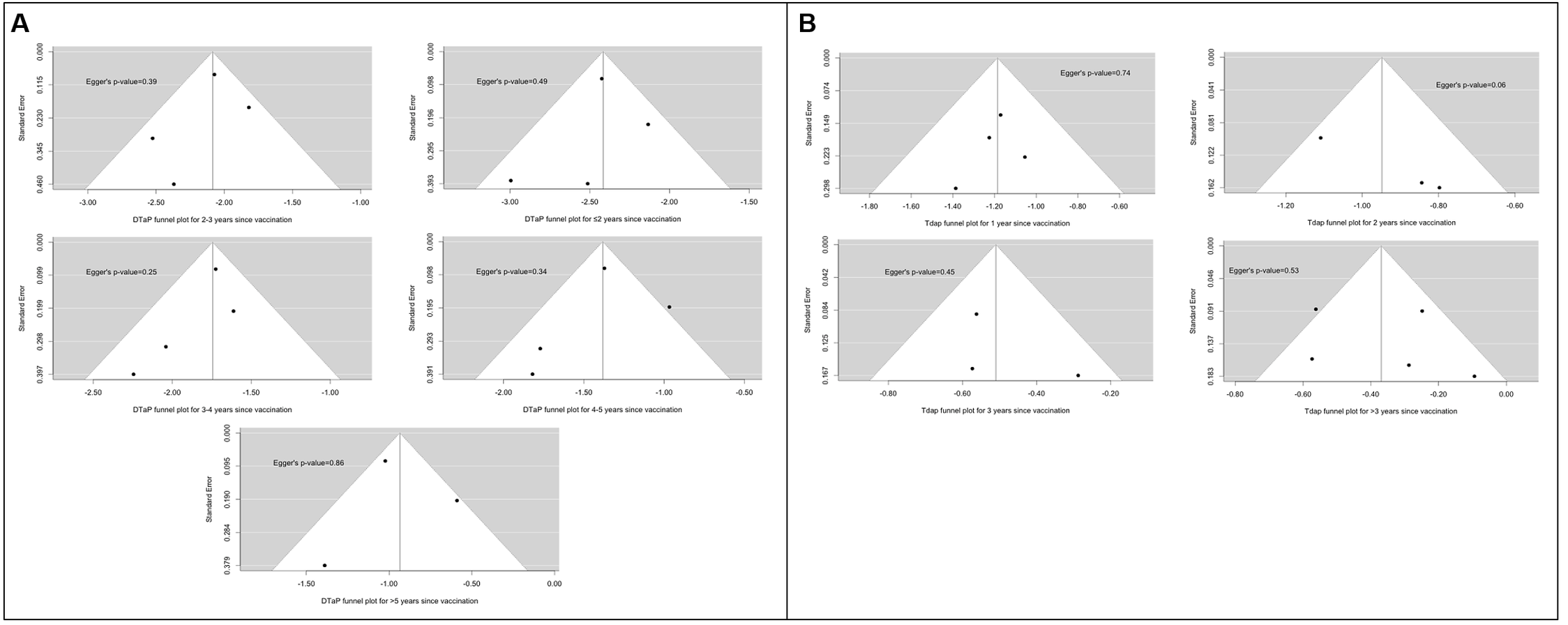

Supplement: S3 Fig — (TIFF) [file pone.0197970.s004.tiff]

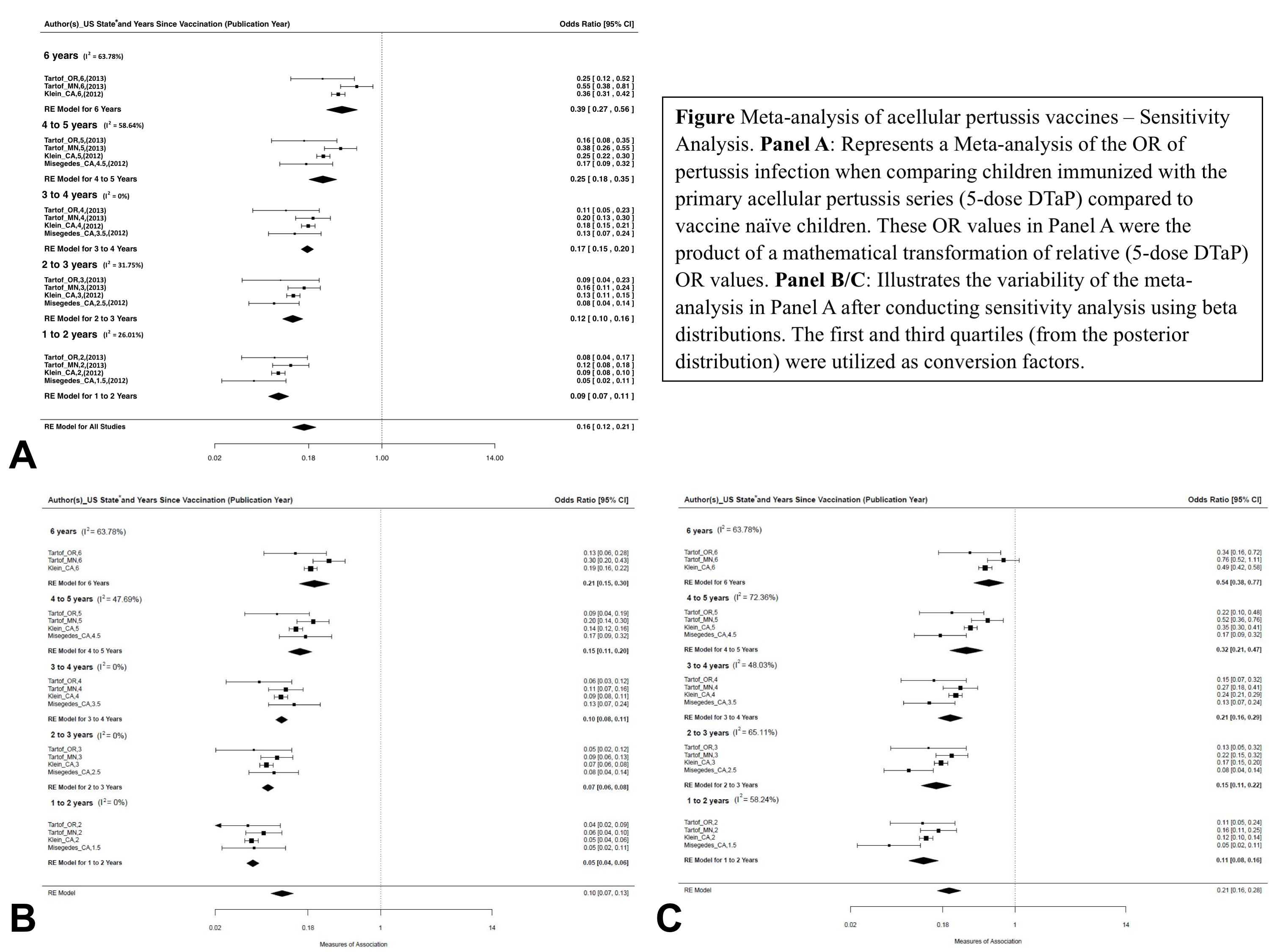

Supplement: S4 Fig — (TIFF) [file pone.0197970.s005.tiff]
